# Intracellular C3 regulates the immune response to infection via NF-κB signaling

**DOI:** 10.1007/s00018-025-05975-4

**Published:** 2025-12-24

**Authors:** Katarzyna Kuska, Serena Bettoni, Frida Mohlin, Maja Chrobak, Vaishnavi Dandavate, Saleh Moradi, Ben King, Kristian Riesbeck, Anna M. Blom

**Affiliations:** 1 Medical Protein Chemistry, Lund University, Malmö, Sweden; 2 Clinical Microbiology, Department of Translational Medicine, Faculty of Medicine, Lund University, Malmö, Sweden

**Keywords:** Complement component C3, Intracellular complement, Inflammation, Toll-like receptors, Cytokines

## Abstract

Complement factor C3 is one of the most abundant proteins in the bloodstream and a central part of the complement system. Upon activation, C3 facilitates bacterial recognition and clearance in the extracellular environment. Initially regarded as a serum effector component, C3 is also emerging as an intracellular protein regulating basic cellular processes. Previously, we reported that intracellular, cytosolic C3 can opsonize bacteria and impact their virulence. In this study, we show that cells lacking C3 exhibit altered gene expression that influences immune responses to infection and inflammation. We observed decreased cytokine secretion in C3-deficient cells, which was rescued by expression of non-canonical, cytosolic C3. Further investigation revealed that C3 deficiency impairs signal transduction within the NF-κB signaling pathway, which is attributed to decreased expression of Toll-like receptors. This effect is reversed in cells expressing cytosolic C3, where receptor expression and pathway activation are restored. Therefore, we propose a novel role of intracellular, cytosolic C3 in shaping immune responses by modulating the expression of receptors critical to pathogen recognition.

**Supplementary Information:**

The online version contains supplementary material available at 10.1007/s00018-025-05975-4.

## Introduction

The complement system is a highly conserved part of the immune system, consisting of over 30 circulating and membrane-bound proteins. It is crucial for the detection and removal of pathogens and harmful self-antigens. Complement activation leads to pathogen opsonization and lysis, as well as the generation of potent proinflammatory molecules [1]. C3 converges the classical, lectin, and alternative activation pathways, making it a central molecule in the complement cascade [2]. During complement cascade activation, C3 is enzymatically cleaved to C3a and C3b. C3a acts as a potent anaphylatoxin, while C3b functions as an opsonin, covalently binding to the pathogen’s surface, leading to phagocytosis and lysis [1, 3].

C3 is among the most prevalent proteins in the blood and is mainly expressed in the liver, although most cell types in the body express certain amounts of C3 [4–8]. Traditionally, C3 has been viewed as a protein operating in the serum, but over the past decade, emerging evidence has shown that it also plays roles inside of the cell, extending beyond its canonical functions in innate immunity. Thus, in T cells, intracellular C3 is activated by cleavage into C3a and C3b that is mediated by cathepsin L. C3a then engages the C3a receptor (C3aR), leading to mTOR activation, which is essential for homeostatic cell survival [9]. It has also been shown that C3 can localize intracellularly when it opsonizes invading pathogens, becoming internalized into the cell along with the pathogen [10, 11].

Our group recently demonstrated two mechanisms by which C3 can localize to the cytosol, including alternative translation and retrotranslocation [12]. Canonical C3 is expressed as pre-pro protein with a signal peptide directing it into the ER and the secretory pathway [13]. However, it can also be translated from the second, non-canonical translation start site directly into the cytosol. We demonstrated that, in human type II alveolar epithelial cells (A549), cytosolic C3 can opsonize bacteria intracellularly. Whole-blood survival of bacteria released from wild-type cells was decreased compared to those from C3-deficient cells, suggesting that deposition of intracellular C3 can increase phagocytosis [12].

In this study, the non-canonical roles of C3 in infection and inflammation were investigated. Since non-hematopoietic cells play a significant role in immune defense, especially in organs like the lungs that are constantly exposed to pathogens [14, 15], we utilized the A549 lung epithelial cell line. Previous studies have shown that intrinsic C3 in the lung is required for protection against acute bacterial pneumonia, as mice deficient only in liver-derived C3 remain protected [16]. Here, we show that C3-deficient A549 cells exhibit reduced cytokine secretion in response to bacterial infection and pathogen-associated molecular patterns (PAMPs), which is restored in cells expressing only cytosolic C3. We found that cells infected with certain bacteria or treated with specific Toll-like receptor (TLR) agonists require intracellular C3 for proper signal propagation in the NF-κB signaling pathway. We demonstrate that this downregulation of the immune response in the absence of C3 may be linked to decreased TLR expression.

## Results

### C3 deficiency induces changes in the lung epithelial cell transcriptome

**Fig. 1:**
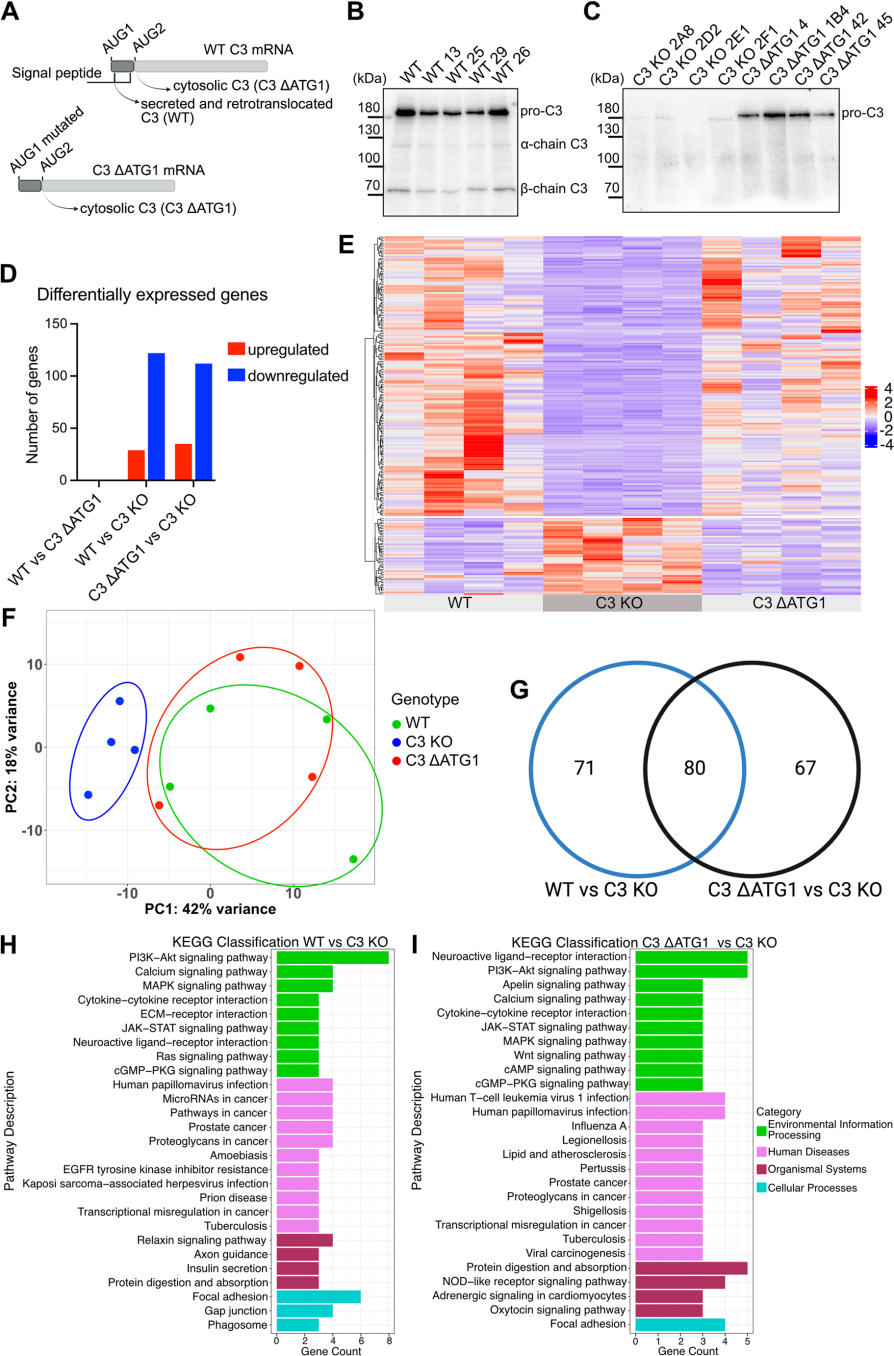
C3 deficiency alters gene expression in human lung epithelial A549 cell line, which is rescued by cytosolic C3. **A**) Three variants of A549 cells expressing C3 (WT), entirely lacking C3 (C3 KO) or expressing only cytosolic C3 (C3 ΔATG1) were used in this study. Western blotting analysis of cell lysates showing the presence of pro-C3 along with mature alpha and beta chains in WT cells (**B**). No C3 was detected in C3 KO cells while C3 ΔATG1 clones expressed only pro-C3 (**C**). **D**) While WT and C3 ΔATG1 cells showed similar gene expression, C3 KO cells had strongly altered gene expression compared to WT and C3 ΔATG1 (padj < 0.05). **E**) Heat map representation of the expression patterns in WT, C3 KO and C3 ΔATG1 clones (4 clones for each genotype) and **F**) Principal component (PC) analysis of RNA-seq data for WT, C3 KO and C3 ΔATG1 clones showed that C3 KO clones cluster separately from WT and C3 ΔATG1 clones. **G**) Venn diagram of DEGs across different genotypes. Summary of KEGG enrichment analysis showing pathways with >2 differentially expressed genes between WT and C3 KO (**H**) and between C3 KO and C3 ΔATG1 (**I**). For analysis of differentially regulated genes, we used the following criteria: pair-wise analysis with padj≤ 0.05 & |log2(foldchange)| ≥ 1

To distinguish between the role of the canonical, secreted C3 and intracellular, cytosolic C3 we utilized previously generated C3-deficient (C3 KO) cells and cells expressing only cytosolic C3 (designated C3 ΔATG1) together with wild-type cells (WT) (Fig. 1A) [12]. Genotypes were verified using Western blotting (Fig. 1B, C). We have previously demonstrated that cytosolic C3 targets cytoinvasive pathogens and delays their vacuolar escape in a lung epithelial cell line (A549) [12]. Consequently, we sought to further investigate the role of intracellular C3 in bacterial infection and inflammation in epithelial cells. To this end, we performed RNA sequencing (RNA-seq) to examine transcriptome patterns in WT, C3 KO and C3 ΔATG1, using 4 individual clones per genotype at basal conditions. Volcano plots of differentially expressed genes confirmed the absence of C3 expression in C3 KO cells, thereby validating their use in the transcriptomic analysis (Fig. S1A, B). A similar number of differentially expressed genes were detected in C3 KO cells compared to the WT (151 genes) and C3 ΔATG1 (147 genes) cells (Fig. 1D). Most of these differentially expressed genes were downregulated in C3 KO cells. Interestingly, no significant changes were observed in WT vs. C3 ΔATG1 cells. This fact was also evident when genes were clustered in the heatmap (Fig. 1E). Principal component analysis (PCA) showed a clear overlap between the WT and C3 ΔATG1 groups, while the C3 KO cells clustered separately (Fig. 1F). These results suggest that cytosolic C3 alone, i.e., C3 ΔATG1, is sufficient to maintain normal transcriptional activity. Although the number of differentially regulated genes in WT vs. C3 KO and C3 ΔATG1 vs. C3 KO was similar, the identity of these genes was distinct. In Fig. 1G, a Venn diagram of the two subsets is presented; 80 genes overlap when the WT vs. C3 KO and C3 ΔATG1 vs. C3 KO are compared, whereas 71 and 67 genes were distinct to WT vs. C3 KO and C3 ΔATG1 vs. C3 KO, respectively. Furthermore, we performed a KEGG pathway analysis and focused on pathways containing two or more differentially expressed genes (DEGs) (Fig. 1H, I). KEGG analysis indicated several pathways that are engaged in infection and inflammation. For example, PI3K-Akt and JAK-STAT signaling pathways were altered in both WT and C3 ΔATG1 cells compared to KO. These pathways play a crucial role in promoting macrophage activation, an essential step in the immune response [17, 18]. Additionally, they can mediate induction of different cytokines, such as MCP-1 and interleukin-6 (IL-6) [19], and they contribute to interstitial lung diseases [20]. Moreover, C3 deficiency affected cytokine-cytokine receptor interaction pathways, suggesting a potential role of C3 in that process as well (Fig. 1H, I). Collectively, these results indicate that C3 deficiency affects multiple pathways and interactions that are vital in the inflammatory response.

### Cytokine secretion from human lung epithelial A549 cells is modulated by cytosolic C3

**Fig. 2:**
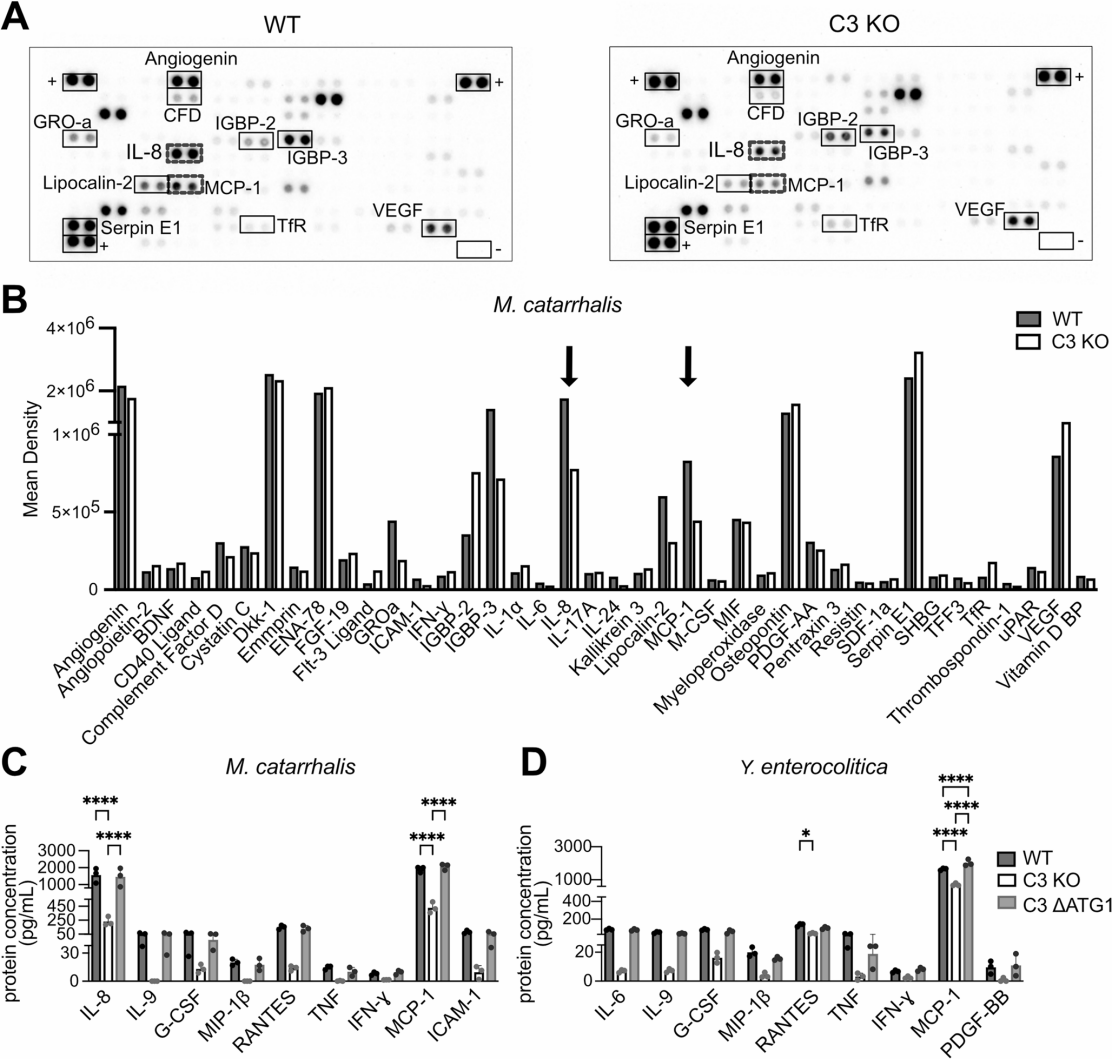
Cytokine secretion from A549 cells is modulated by the presence of C3. XL cytokine array analysis of supernatants from WT and C3 KO A549 cells infected with *M. catarrhalis* shows lower secretion from C3 KO cells. Reference spots and negative controls are indicated by + and –, respectively (**A**) with quantification in (**B**), and black arrows indicating IL-8 and MCP-1, as differentially secreted cytokines. Bio-Plex Pro human cytokine 27-plex assay with supernatants from WT, C3 KO and C3 ΔATG1 cells infected with *M. catarrhalis* (**C**) or *Y. enterocolitica* (**D**) shows lower secretion from C3 KO cells that is rescued in C3 ΔATG1. Bars represent the average of three biological repetitions indicated with symbols. Two-way ANOVA with Tukey´s multiple comparison test, matched measures within the experiment, *p < 0.05, ****p < 0.0001

Since DEGs identified between C3-deficient and C3-expressing cells seem to relate to cytokine-regulated immune responses, we first investigated cytokine and chemokine secretion from infected A549 cells, using an array for a broad range of secreted proteins. Supernatants collected from WT and C3 KO cells after infection with the respiratory pathogen *Moraxella catarrhalis* were analyzed using the Human XL Cytokine Array. This analysis indicated multiple proteins with reduced secretion in C3-deficient cells. Among these, IL-8 and MCP-1 were the most interesting targets as they are involved in a pro-inflammatory response to bacteria (Fig. 2A, B) [21, 22]. Additionally, as KEGG pathway analysis (Fig. 1H, I) also indicated several pathways involved in viral infections, we investigated interferon β secretion; however, no detectable levels were observed (data not shown).

Cytokine and chemokine secretion was further explored in a multiplex array containing a panel of 27 analytes. Wild-type and C3-deficient epithelial cells in addition to cells expressing only the cytosolic form of C3 were infected with gram-negative bacteria (*M. catarrhalis* or *Yersinia enterocolitica*) for 1.5 h, followed by analyses of supernatants after 18 h. Interestingly, in C3 KO cells, several cytokines and chemokines (IL-6, IL-8, IL-9, G-CSF, MIP-1β, TNF, RANTES, IFN-γ, MCP-1, ICAM-1, PDGF-BB) were secreted at a lower level (Fig. 2C, D). In parallel with *M. catarrhalis* and *Y. enterocolitica*, incubation with *Haemophilus influenzae* or *Neisseria gonorrhoeae* showed the same profiles (Fig. S2A, B). Intracellular survival of bacteria was not modulated by the presence of C3 and did not correlate with cytokine secretion (Fig. S2C). Since C3 ΔATG1 cells exhibited comparable levels of secreted cytokines and chemokines as WT epithelial cells, cytosolic C3 may be involved in the expression and consequently secretion of these inflammatory markers.

### Cytosolic C3 contributes to cytokine expression and secretion after stimulation of different receptors

**Fig. 3:**
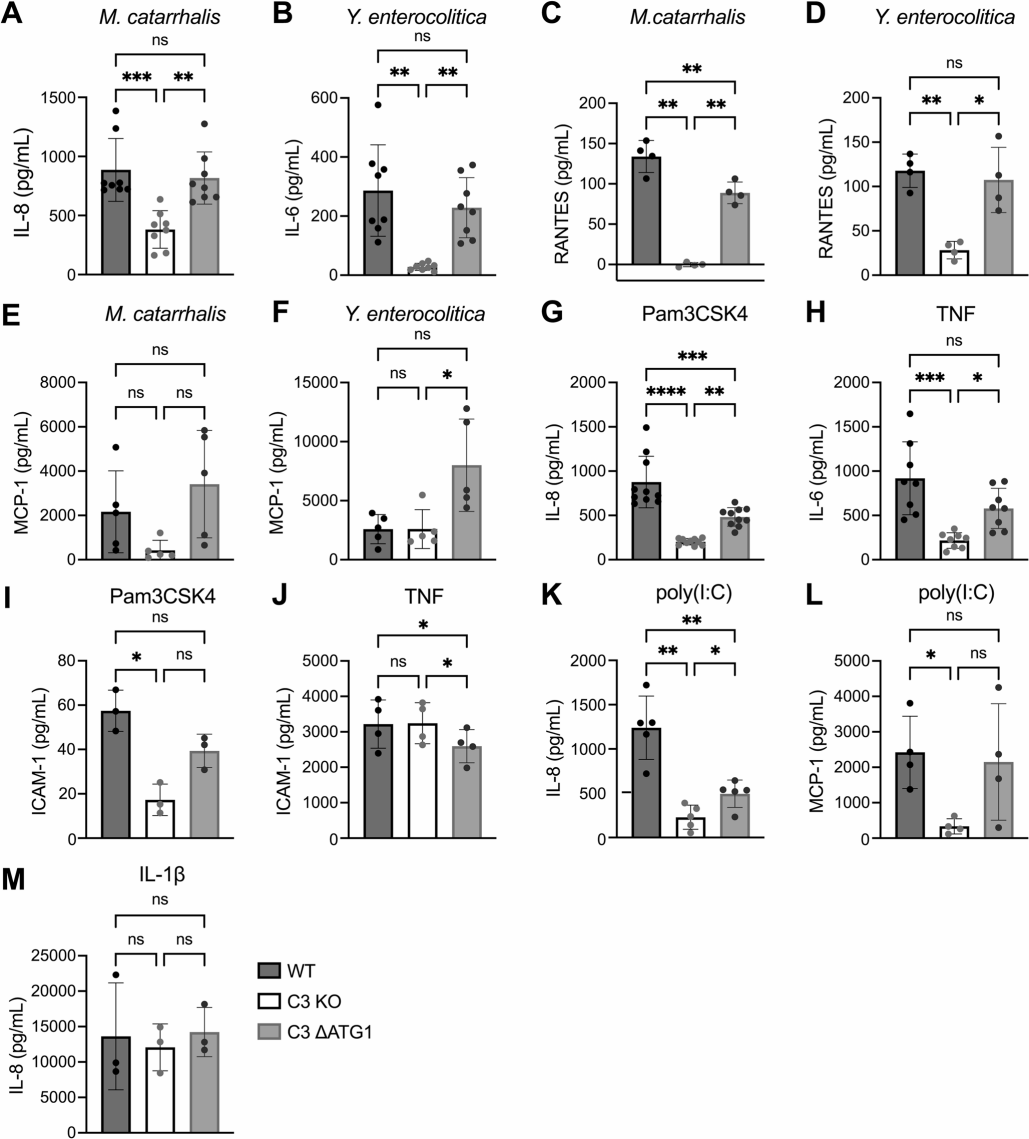
Cytosolic C3 contributes to cytokine release after stimulation of different receptors. (**A**-**F**) Levels of cytokine secretion measured by ELISA in supernatants from WT, C3 KO and C3 ΔATG1 A549 cells after infection with bacteria are reduced in cells lacking C3. IL-8 secretion after infection with *M. catarrhalis* (**A**), IL-6 after infection *Y. enterocolitica* (**B**), RANTES secretion after infection with *M. catarrhalis* (**C**), RANTES secretion after infection with *Y. enterocolitica* (**D**), MCP-1 secretion after infection with *M. catarrhalis* (**E**), and MCP-1 secretion after infection with *Y. enterocolitica* (**F**). (**G**-**M**) Levels of cytokine secretion in supernatants from WT, C3 KO and C3 ΔATG1 A549 cells are reduced in cells lacking C3 after stimulation of TLR or TNFR, but not after IL-1R stimulation. IL-8 secretion after treatment with Pam3CSK4 (**G**), IL-6 secretion after treatment with TNF (**H**), sICAM-1 release after treatment with Pam3CSK4 (**I**), sICAM-1 release after TNF treatment (**J**), IL-8 secretion after poly(I: C) treatment (**K**), MCP-1 secretion after poly(I: C) treatment (**L**), and IL-8 secretion after IL-1β treatment (**M**). Results are shown as mean ± SD of independent experiments. Each dot represents an average of at least three clones in one biological repeat. The cytokine secretion levels of untreated cells were subtracted from the values of treated cells. One-way ANOVA with Tukey´s multiple comparison test, matched measures within experiment for C-F and I-M. *p < 0.05, **p < 0.01, ***p < 0.001, ****p < 0.0001, ns: not significant

To confirm the results from protein arrays, we included specific ELISAs targeting cytokines and chemokines in our experimental set ups. Results showed that absence of C3 significantly impaired IL-8 or IL-6 secretion when cells were infected with *M. catarrhalis* or *Y. enterocolitica* respectively (Fig. 3A, B). In addition, RANTES appeared to be modulated by both bacterial species (Fig. 3C, D). Similarly, cells expressing cytosolic C3 secreted inflammatory proteins at a level comparable to WT epithelial cells. The chemokine MCP-1 was also modulated by C3. Interestingly, MCP-1 exhibited higher secretion in C3 ΔATG1 cells compared to WT and C3 KO cells, albeit only statistically significant in the presence of *Y. enterocolitica* (Fig. 3E, F). Release of soluble ICAM-1 (sICAM-1) was also investigated, given that it is an important marker of inflammation. Significantly lower levels of sICAM-1 were observed in C3-deficient cells infected with *M. catarrhalis* (Fig. S3A). The opposite effect was however observed when cells were infected with *Y. enterocolitica*; here WT and C3 ΔATG1 cells had a lower sICAM-1 release compared to C3 KO cells (Fig. S3B). Given that bacteria stimulate cells through multiple pattern recognition receptors (PRR) with different PAMPs and knowing that IL-6, IL-8, RANTES and ICAM-1 are expressed after activation of intracellular signaling pathways, we therefore explored whether the effect of C3 on cytokine production was receptor-specific. Using both Pam3CSK4 and TNF, which bind and activate the TLR1/2 and TNF receptor, respectively, we observed a similar pattern of modulation for IL-8 and IL-6 secretion, i.e., C3-deficient cells had significantly lower levels of cytokines in comparison to WT, and cytosolic C3 was partially rescuing the wild-type phenotype in C3 ΔATG1 cells (Fig. 3G, H). sICAM-1 was also modulated when Pam3CSK4 was used (Fig. 3I). Interestingly, when cells were stimulated with TNF, a pattern similar to *Y. enterocolitica* infection was observed; C3-deficient cells secreted comparable levels of sICAM-1 to WT cells, and C3 ΔATG1 had a lower secretion than these two (Fig. 3J). C3 deficiency also hampered IL-8 and MCP-1 secretion in response to poly(I:C), which stimulates TLR3, the only TLR that activates the signaling pathway independent from MyD88, a main adaptor protein in NF-κB pathway (Fig. 3K, L). Moreover, cells were treated with IL-1β, another activator of the NF-κB cascade that triggers the interleukin-1 receptor (IL-1R). There was no difference in IL-8 secretion between WT, C3 KO and C3 ΔATG1 cells (Fig. 3M). Next, we measured IL-8 and sICAM-1 levels in cell lysates following Pam3CSK4 treatment and observed similar downregulation in C3 KO cells compared to WT and C3 ΔATG1 cells (Fig. S3C, D). Those results suggest that cytokine expression, rather than secretion, is affected. Furthermore, we tested how IL-1β and TNF treatment influence C3 expression and processing in A549 cells, since those cytokines are known C3 upregulators [23]. Indeed, both IL-1β and TNF increased C3 expression in WT and C3 ΔATG1 cells (Fig. S3E, F), however, increased processing of C3 into α and β chains was not observed. Expression of molecules such as IL-8 and ICAM-1 is induced by NF-κB activation; however, the underlying mechanisms and stimuli can differ between them [24, 25], leading to distinct responses to various stimuli depending on the presence or absence of C3. In conclusion, our results support the hypothesis that C3, specifically in its cytosolic form, plays an important role in the modulation of cytokine release. Our findings also show that this role is not attributed to one specific receptor but instead seems to influence secretion triggered by different stimuli.

### Transcription of *IL6* and *CXCL8* genes is partially dependent on intracellular C3

To define the molecular mechanisms by which cytosolic C3 regulates cytokine response, we then quantified mRNA levels of IL-6 (*IL6*) and IL-8 (*CXCL8*) in epithelial cells incubated with bacteria (*M. catarrhalis* or *Y. enterocolitica*) and compared to stimulation with TNF and Pam3CSK4.

**Fig. 4:**
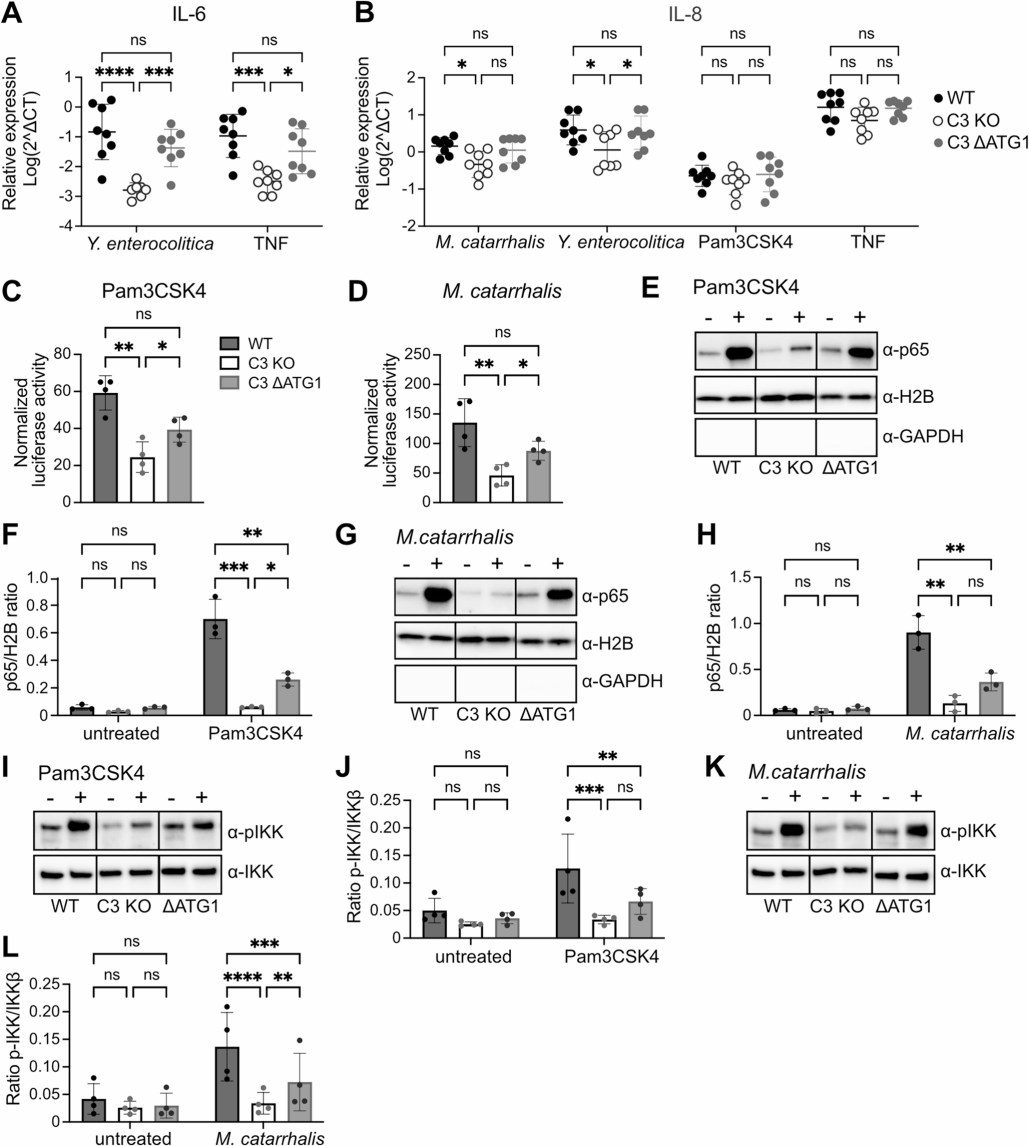
Cytokine gene transcription and NF-κB pathway activation are dependent on cytosolic C3. mRNA levels for IL-6 are decreased in C3 KO A549 cells compared to WT and C3 ΔATG1 cells after infection with *Y. enterocolitica*, or treatment with TNF (**A**). Similar is observed for mRNA levels for IL-8 after infection with *M. catarrhalis* or *Y. enterocolitica*, however treatment with TNF or Pam3CSK4 does not reach significance (**B**). NF-κB activation assessed using luciferase reporter assay is decreased in C3 KO compared to WT and C3 ΔATG1 cells after treatment with Pam3CSK4 (**C**) or infection with *M. catarrhalis* (**D**). Representative Western blot showing NF-κB (p65 subunit) translocation to the nucleus (**E**) with quantification in (**F**). C3 KO cells are characterized by diminished translocation of p65 to the nucleus compared to WT and C3 ΔATG1;- and + indicate no treatment and treatment with Pam3CSK4, respectively. A similar result was obtained upon infection with *M. catarrhalis*, a representative blot of nuclear fractions shown in (**G**) with quantification in (**H**). Representative Western blot analysis showing decreased phosphorylation of IKK in lysates from C3 KO cells compared to WT; - and + indicate no treatment and treatment with Pam3CSK4 (**I**) or *M. catarrhalis* (**K**) with quantification in (**J**) and (**L**), respectively. Each dot represents one biological repeat for one clone (**A**, **B**) or an average of two clones in one biological repeat (**C**-**L**). One-way (**C**, **D**) or Two-way (**A**, **B**, **F**, **H**, **J**, **L**) ANOVA with Tukey´s multiple comparison test, matched measures within the experiment. *p< 0.05, **p < 0.01, ***p < 0.001, ns: not significant

*IL6* transcripts were generally undetectable for epithelial cells infected with *M. catarrhalis* or treated with Pam3CSK4. However, aligning with our previous ELISA results, mRNA levels of *IL6* in *Yersinia*-infected or TNF-stimulated cells and *CXCL8*, for bacterial infection were significantly higher in C3-expressing cells in comparison to C3-deficient cells and similar trend was observed for treatments with Pam3CSK4 and TNF (Fig. 4A, B). The quantity of transcripts obtained from C3 ΔATG1 cells was similar to those isolated from WT cells, which suggests that optimal cytokine gene transcription requires the presence of the intracellular, cytosolic form of C3 in human cells.

### NF-κB transcriptional activity is impaired in C3-deficient cells

The transcriptional activity of NF-κB regulates various aspects of innate and adaptive immune responses, including cytokine and chemokine expression [26]. Since we observed decreased cytokine expression and secretion in C3-deficient cells, we studied the role of C3 in NF-κB activation. A dual luciferase reporter assay was included to monitor NF-κB activation in A549 epithelial cells infected with bacteria or treated with Pam3CSK4 or LPS, which stimulate TLR1/2 and TLR4, respectively. C3 KO cells had a significantly lower NF-κB activation compared to WT and C3 ΔATG1 cells following treatment with Pam3CSK4 or infection with *M. catarrhalis* or LPS (Fig. 4C, D, S3G). Notably, cells expressing only cytosolic C3 were able to partially rescue the wild-type phenotype, suggesting a novel, non-canonical role of C3 in the NF-κB signaling cascade.

### NF-κB translocation to the nucleus is impaired in C3-deficient human lung epithelial A549 cells

NF-κB is an inducible transcription factor that is sequestered in the cytoplasm and translocates into the nucleus upon activation, enabling a rapid and robust response [27]. Thus, based on these facts, we investigated translocation of p65, one of the NF-κB subunits, into the nucleus. Cells were treated with Pam3CSK4 or infected with *M. catarrhalis*, subjected to subcellular fractionation after 60 min, followed by analysis of p65 in nuclear fractions (Fig. 4E-H). In cells deficient of C3 almost no translocation was observed, in stark contrast to WT cells where significantly more p65 was found in the nucleus after treatment and bacterial infection (Fig. 4F, H). In case of C3 ΔATG1 cells, translocation was higher compared to C3 KO cells, but it reached statistical significance only upon Pam3CSK4 treatment (Fig. 4E, F). In the untreated cells, barely any p65 was found in the nucleus and no difference was observed between the genotypes (Fig. 4F, H). Our results suggest that C3 deficiency in A549 cells leads to impaired NF-κB translocation into the nucleus which in turn leads to decreased cytokine expression. Notably, NF-κB translocation was partially rescued in cells expressing only cytosolic C3 again suggesting a novel role for this variant of C3.

### Intracellular, cytosolic C3 plays a role in signal transduction in NF-κB signaling pathway

To further investigate the NF-κB signaling pathway, we focused on a central regulator of NF-κB signaling, i.e., the inhibitor of κB (IκB) kinase (IKK). IKK phosphorylation was measured as this step is essential for signal transduction in the pathway. When the pathway was activated through TLR1/2 with Pam3CSK4, C3-deficient cells exhibited decreased IKK phosphorylation compared to cells expressing C3, however, the difference was not statistically significant in cells expressing only cytosolic C3 (Fig. 4I, J). Infection with *M. catarrhalis* showed the same result and C3 ΔATG1 cells had a significantly higher IKK phosphorylation as compared to C3 KO cells (Fig. 4K, L). This indicates that C3 deficiency alters signal transduction within the NF-κB signaling cascade and that cytosolic C3 is sufficient to partially restore the activation.

### C3-deficient cells are characterized by lower expression of TLRs

**Fig. 5:**
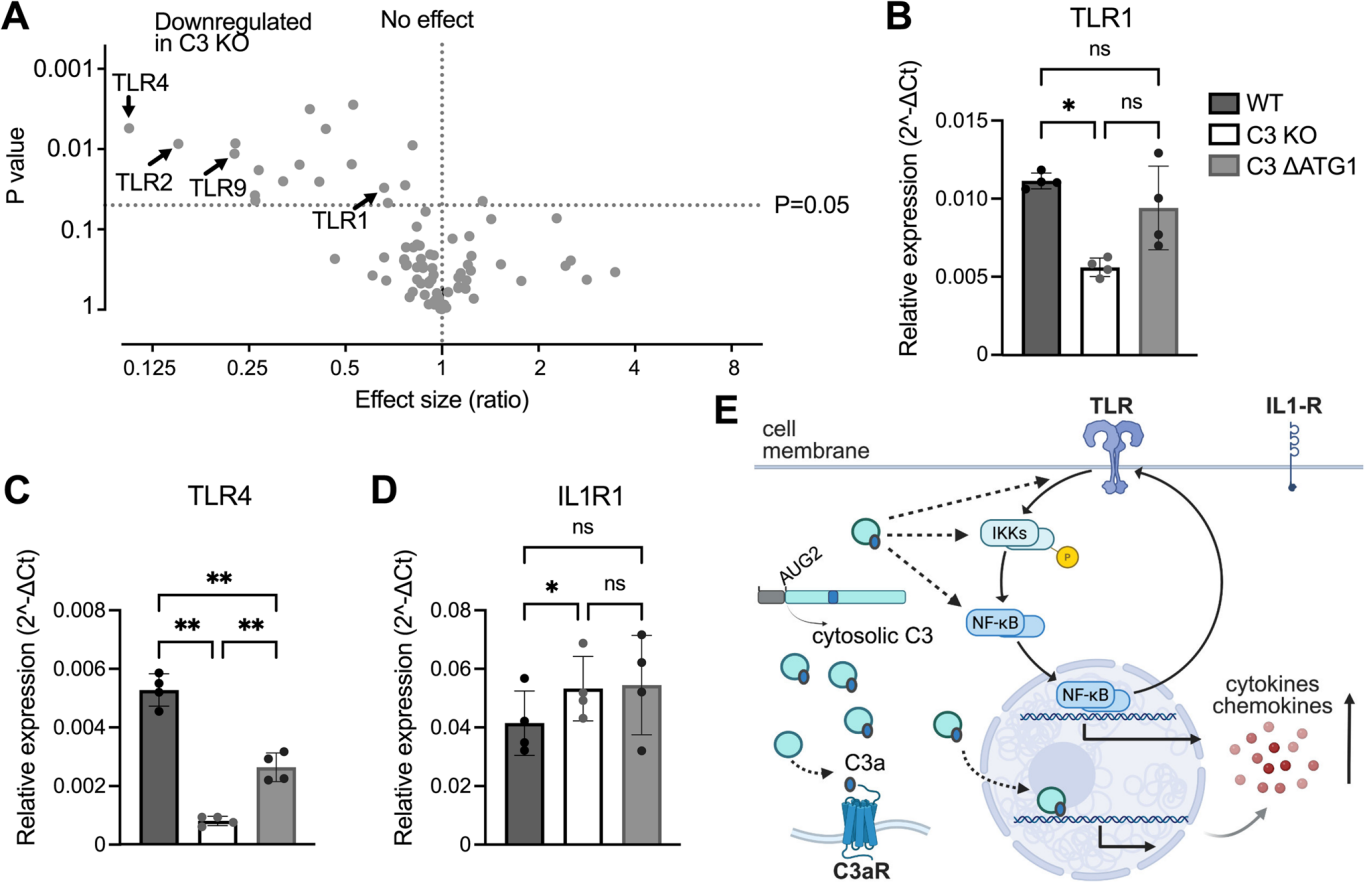
C3 KO cells are characterized by lower expression of TLRs. mRNA expression levels of proteins involved in NF-κB pathway were analyzed using RT-PCR array. Altered TLRs are indicated with arrows (**A**). mRNA levels of TLR1 (**B**), TLR4 (**C**) and IL1R1 (**D**) were measured using Q-PCR. C3 KO cells showed downregulation of TLR1 and TLR4 compared to WT and C3 ΔATG1, while levels of IL-1R remained unchanged. Schematic illustrating potential mechanisms through which cytosolic C3 might influence gene transcription and cytokine release, possibly via interactions with components of the TLR pathway in the cytosol or nucleus, or through a role for intracellular C3aR (**E**). In B-D each dot represents one biological repeat for two clones. One-way ANOVA with Tukey´s multiple comparison test, matched measures within experiment. *p < 0.05, **p < 0.01, ns: not significant

Since we observed altered signal transduction in the NF-κB pathway in C3-deficient epithelial cells, we decided to study in detail the gene expression of various proteins involved in this pathway. To this end we included an RT-PCR array to assess expression levels of various mRNAs related to the NF-κB cascade. Most of the down-regulated mRNAs were found in C3-deficient cells, which aligned with the RNA-seq analysis (Fig. 1). Among the down-regulated mRNAs we found four TLRs, i.e., *TLR1*, *TLR2*, *TLR4* and *TLR9* (Fig. 5 A). We further confirmed that *TLR1* and *TLR4* were significantly down-regulated in C3 KO cells, and cytosolic C3 was enough to at least partially rescue the wild-type phenotype (Fig. 5B, C). Interestingly, *IL1R1* levels were not decreased in C3-deficient cells; on the contrary, expression appeared to be lower in wild-type cells compared to C3 KO cells (Fig. 5D). This finding explained why IL-1β treatment did not modify cytokine secretion from C3-deficient cells (Fig. 3 M), especially since NF-κB pathway activated by TLRs or IL1-R converges upstream in the signaling pathway (Fig. 5 E), Notably, changes in TLR expression were detected in untreated cells, therefore suggesting that C3 plays a role in regulating the immune responses by modulating gene expression even before any threat is encountered.

## Methods

### Cell culture

The wild-type (WT) human lung epithelial A549 cells were from the American Type Culture Collection (ATCC). C3 mutant clones were generated using the CRISPR/Cas9 gene editing technology, as previously described [12]. These included knockouts with a full C3 deletion (C3 KO), created by deleting the genomic region spanning exons 4 through 13 of the *C3* locus, and C3 ΔATG1 clones with frameshift mutation within the signal peptide. Only the WT cells had both secreted and cytosolic C3, whereas the C3 ΔATG1 clones only expressed the cytosolic pro-C3 form. A549 clones were maintained in DMEM (Cytiva) with 10% heat-inactivated fetal bovine serum (FBS, Gibco). All cells were tested regularly for *Mycoplasma* contamination (Eurofins Genomics) and cultured at 37 °C in 5% CO_2_. For bacterial infection and agonist treatment, cells were seeded into a 24-well Corning^®^ CellBIND^®^ plate at a density of 1.2 × 10^5^ cells per well, one day before the experiment.

### RNA sequencing

Four individual clones each of WT, C3 ΔATG1 and C3 KO A549 cells were seeded onto 6-well Corning^®^ CellBIND^®^ plates at a density of 5 × 10^5^ cells per well, one day before the experiment. Total RNA was isolated using RNeasy Plus Mini Kit (Qiagen). RNA quantity and quality were measured using a Biodrop spectrophotometer and 2200 Tapestation (Agilent Technologies). Samples were then subjected to commercial RNA-seq analyses (Novogene). The data from this study have been submitted to the GEO repository with accession number GSE301708, and the method is described in detail in the Supplementary Material. DEGs, KEGG pathways, and a list of overlapping genes can be found in online resources.

### Bacterial infection of A549 cells

*Yersinia enterocolitica* CCUG4586 (purchased from the Culture Collection University of Gothenburg, CCUG), *Moraxella catarrhalis* RH4 [28], *Haemophilus influenzae* 86-028NP [29] and *Neisseria gonorrhoeae* SA94 [30] were cultured overnight (*Y. enterocolitica* in LB, *M. catarrhalis* in brain heart infusion broth (BHI), *H. influenzae *in BHI + hemin (2 µg/mL), *N. gonorrhoeae* on chocolate blood agar plates). Next day, bacteria were diluted (*Y. enterocolitica* − 1:20, *M. catarrhalis* and *H. influenzae* to OD_600nm_ = 0.1, *N. gonorrhoeae* was passed on a new plate) and cultured again at 37 °C to generate a logarithmic growth-phase culture. After 3–5 h bacteria were collected, washed with PBS, and resuspended in Opti-MEM at a concentration of 1 × 10^8^ CFU/mL. Three individual clones each of WT, C3 ΔATG1 and C3 KO A549 cells were thereafter infected at a Multiplicity of Infection of 100. Plates, which contained human cells and bacteria, were spun down (500 x g, 5 min) to promote contact. After 1.5 h of infection medium was replaced with Opti-MEM containing 200 µg/mL of gentamicin. The plates were incubated for an additional 18 h. Supernatants were collected and frozen for cytokine measurement. Cells that remained on the plate were washed twice with PBS and detached with 200 µL of 0.05% trypsin (Cytiva) for 5 min at 37 °C, 5% CO_2_. After adding 300 µL of DMEM, cells were harvested, centrifuged at 300 x g for 5 min, and cell pellets were stored at −80 °C for subsequent RNA isolation and gene expression analysis.

### Agonist treatment

Two individual clones each of WT, C3 ΔATG1 and C3 KO A549 cells were treated with either 1 µg/mL Pam3CSK4 (InvivoGen #tlrl-pms), 1 µg/mL of LPS (InvivoGen #tlrl-eklps), 0.1 ng/mL IL-1β (Mabtech #3415–1 H-6), 10 ng/mL TNF (ImmunoTools #11343015) or 100 ng/mL poly(I: C) (InvivoGen #tlrl-picw) and incubated for 24 h before the supernatants were collected and cells were lysed in lysis buffer containing 150 mM NaCl, 50 mM Tris, pH 7.5, 1% NP40 and 0.5% sodium deoxycholate, supplemented with Halt™ Protease and Phosphatase Inhibitor Cocktail, EDTA-free (100X) (Thermo Scientific™, #78441). Working solutions of each agonist were filtered through a 0.2 μm filter before addition to the cells.

### Cytokine profiling and ELISAs

Collected cell supernatants and lysates were used to detect cytokines using Proteome Profiler Human XL Cytokine Array Kit (R&D systems #ARY022B), Bio-Plex Pro human cytokine 27-plex assay (Bio-Rad #M500KCAF0Y) or commercial ELISAs from R&D systems (Human IL-6 DuoSet ELISA # DY206-05, Human IL-8 DuoSet ELISA # DY208, Human CCL2/MCP-1 DuoSet ELISA #DY279, Human CCL5/RANTES DuoSet ELISA #DY278-05, and Human ICAM-1/CD54 DuoSet ELISA #DY720-05), following the manufacturer’s protocols. The assay was performed on 96-well MicroWell™ MaxiSorp™ flat-bottom plates. Reagents not provided within a kit but used in the experiment included Tween 20™ (Thermo Scientific), bovine serum albumin (BSA, Sigma), and DPBS. The signal was developed using TMB ONE ELISA substrate (Kementec), and the reaction was stopped with 0.5 M H_2_SO_4_ (Honeywell). Absorbance after signal development was determined using BioTek Cytation5 Cell Imaging Multimode Reader set to 450 nm. Wavelength correction was set at 680 nm, and the results are shown as subtracted levels of uninfected cells.

### RNA isolation, cDNA synthesis, NF-κB qPCR array and RT-qPCR

RNA from two individual clones each of WT, C3 ΔATG1 and C3 KO A549 cells was extracted using the RNeasy Plus Mini kit (Qiagen), according to the manufacturer’s protocol. RNA quality and integrity were verified on RNA Nano Chips (Agilent RNA 6000 Nano Reagents) compatible with Agilent 2100 Bioanalyzer System (Agilent Technologies). cDNA was synthesized using oligo-dT primers and SuperScript IV (Invitrogen). For NF-κB qPCR array a predesigned panel was used (Bio-Rad). For qPCRs specific probes from Thermo Scientific™ were used: IL-6 (Hs00174131_m1), IL-8 (Hs00174103_m1), TLR1 (Hs00413978_m1), TLR4 (Hs00152939_m1), IL1R1 (Hs00991010_m1), HPRT (Hs99999909_m1) and quantitative PCR was performed with ViiA7 Real-Time PCR system (Thermo Fisher).

### SDS-PAGE and Western blotting

Cells were lysed in 150 mM NaCl, 50 mM Tris, pH 7.5, 1% NP40 and 0.5% sodium deoxycholate, supplemented with Halt™ Protease and Phosphatase Inhibitor Cocktail, EDTA-free (100X) (Thermo Scientific™, #78441). For the detection of C3 in A549 clones, C3 was immunoprecipitated from cell lysates after overnight incubation using anti-human C3c antibody (Dako #A0062), coupled to protein A Dynabeads (Thermo Fisher Scientific #10002D). For analysis of NF-κB p65 translocation to the nucleus, cells were fractionated using ProteoExtract^®^ Subcellular Proteome Extraction Kit (Calbiochem #539790), according to the manufacturer’s instructions. Samples were prepared by mixing with reducing 3x Laemmli Buffer (containing 5% SDS and 20 mM DTT) and then subjected to SDS-PAGE on a gradient 4–20% Mini-PROTEAN^®^ TGX™ Precast Protein Gel (Bio–Rad). Then, proteins were transferred onto PVDF membranes (Trans-Blot Turbo Mini 0.2 μm PVDF Transfer Pack, Bio–Rad) using Trans-Blot^®^ Turbo™ Transfer System (Bio–Rad). Membranes were blocked using either Quench (50 mM Tris, pH 8.0, 150 mM NaCl, 0.1% Tween 20, 3% fish gelatin (Norland Products)) for detection of NF-κB p65 and GAPDH or EveryBlot blocking buffer (Bio-Rad) for detection of phospho-IKKα/β, IKKβ and histone H2B. The primary antibodies used for specific protein detection on the membranes were against human C3 (Calbiochem #204869), NF-κB p65 (Cell Signaling Technology #8242), phospho-IKKα/β (Cell Signaling Technology #2697), IKKβ (Cell Signaling Technology #8943), GAPDH (Abcam #ab8245), and histone H2B (Abcam #ab1790). Corresponding HRP-conjugated secondary antibodies were thereafter added, and the membranes were developed using enhanced chemiluminescence.

### NF-κB activation - Luciferase reporter assay

Two clones each of WT, C3 KO and C3 ΔATG1 A549 cells were seeded in 24-well plates at 7 × 10^5^ cells per well. After 24 h, the cells were transfected with 500 ng of a vector containing a NF-κB response element that drives transcription of firefly luciferase (pGL4.32[luc2P/NF-κB-RE/Hygro], Promega) and 0.7 ng of a vector constitutively expressing Renilla luciferase (pIS2-Renilla, Addgene). After 48 h, cells were infected with *M. catarrhalis* or treated with Pam3CSK4 as described above. After 5 h, cells were washed with PBS and lysed with passive lysis buffer (100 µL per well, 15 min in RT with gentle shaking). Lysates were frozen, and luminescence was measured using Cytation5 reader.

### Statistical analysis

GraphPad Prism version 10.4 was used to perform graph generation and statistical analyses. P-values < 0.05 denoted statistical significance and are displayed as *P < 0.05, **P < 0.01, ***P < 0.001 or ****P < 0.0001. Comparisons were made using one-way or two-way ANOVA with Tukey´s multiple comparison test, and the results are presented as mean with standard deviation.

## Discussion

Apart from its canonical roles in the immune system in serum, C3 also plays non-canonical roles inside of the cell and the existence of intracellular complement is now becoming widely recognized. In beta cells, cytosolic C3 acts as a protective factor against IL-1β-induced cytotoxicity by interacting with Fyn-related kinase (FRK) [31]. Further, C3 interacts with ATG16L1, a key autophagy protein, promoting beta cell homeostasis [32]. The cytosolic interaction between C3 and ATG16L1 also contributes to increased xenophagy of bacteria [10]. Additionally, reinternalized C3 can be sensed inside non-immune cells, leading to activation of the transcription factors, including NF-κB, thus inducing expression of pro-inflammatory cytokines [11]. In lung epithelial cells, cytosolic C3 was shown to detect and opsonize intracellular bacteria and delay their escape to the cytosol [12]. Those novel functions of C3 prompted us to further investigate its roles in inflammation and bacterial infection that go beyond canonical opsonization. As a result, this study identifies cytosolic C3 as a regulator of innate immune responses in epithelial cells by modulating NF-κB signaling and TLR expression.

We observed that C3 deficiency in an epithelial cell line resulted in altered gene expression, primarily characterized by broad downregulation. Many of the differentially expressed genes were associated with pathways central to immune responses during infections, particularly those involved in cytokine secretion. Notably, the cells analyzed by RNA-Seq were neither infected nor otherwise stimulated, suggesting a mechanism of action for C3 distinct from its known role as an opsonin. Further investigation revealed reduced cytokine secretion in C3-deficient cells following both bacterial infection and stimulation with various receptor agonists. Importantly, re-expression of cytosolic C3 alone is sufficient to restore both transcriptional activity and cytokine output, establishing cytosolic C3 as a modulator of epithelial innate immunity. These results suggest that cytosolic C3 contributes to the basal transcriptional priming of innate immune receptors, enabling robust and rapid responses upon pathogen challenge.

Cytokines play a crucial role in inflammation by increasing vascular permeability and promoting the recruitment of immune cells [33]. Epithelial cells, beyond serving as a physical barrier, also contribute to immune defenses by producing cytokines [34]. Therefore, reduced cytokine secretion in A549 cells lacking C3 indicates impaired protective inflammatory function, which may translate to a compromised immune response to infections. Mechanistically, our data indicate that the absence of C3 leads to impaired phosphorylation of IKK, reduced translocation of the NF-κB subunit p65 into the nucleus and diminished transcription of key cytokines such as IL-6 and IL-8. These effects are observed in response to both bacterial infection and stimulation with TLR agonists (e.g., Pam3CSK4, LPS, poly(I:C)), suggesting a generalized deficit in PRR-mediated NF-κB activation. The fact that IL-1β stimulation, which signals through IL-1R rather than TLRs, elicited normal cytokine responses in C3-deficient cells further supports the idea that the regulatory role of cytosolic C3 occurs upstream of NF-κB, likely at the level of PRR expression. Supporting this, we found that several TLR genes were downregulated in C3-deficient cells, while IL-R1 expression was lower in wild-type cells. Thus, the reduced immune response in C3-deficient cells was ultimately due to diminished expression of TLRs – a defect that was reversed by expression of cytosolic C3. Although the NF-κB pathway can be activated by extracellular complement via membrane attack complex (MAC) formation, this occurs through a non-canonical route under specific conditions and is not relevant here [35]. Since altered cytokine secretion was also observed following receptor stimulation alone, MAC involvement can be excluded. These findings instead support a non-canonical, intracellular role for C3 in regulating signal transduction.

Seeking additional validation for the obtained results, we turned to Ingenuity Pathway Analysis (IPA), a powerful tool that allows prediction of upstream regulators and other biological interactions [36]. Consistent with our results, IPA highlighted TNF and NF-κB complex among top 20 upstream regulators inhibited in C3-deficient cells when compared with both WT and C3 ΔATG1 and TLR3 in C3 KO vs. WT cells (Supplementary Tables 1 and 2).

Complement and TLRs are both essential components of the host’s first line of defense and are rapidly activated upon pathogen recognition. Their functional crosstalk likely evolved as a part of coevolution between host and pathogen but remains incompletely understood. Anaphylatoxins C3a and C5a produced during complement activation, act as potent chemoattractants that recruit TLR-expressing immune cells to the site of infection. Simultaneously, TLR activation upregulates the expression of complement components, such as C3 and C5 [37, 38], creating a positive feedback loop that amplifies inflammation. Moreover, signaling via C3aR and C5aR, triggered by C3a or C5a, respectively, can synergize with TLR pathways to enhance transcription factor activation and increase proinflammatory cytokines secretion [39]. Our data suggest that cytosolic C3 contributes to cytokine production by promoting the expression of TLRs. Although the specific C3 fragment responsible for this effect remains unidentified, the known synergy between C3aR and TLRs raises the possibility that intracellular C3aR may be involved. Alternatively, a direct or indirect interaction between intracellular C3, TLRs and components of the NF-κB cascade cannot be excluded.

Previous work from our group demonstrated that intracellular C3 can localize to the nucleus and associate with chromatin, influencing gene expression and function in B lymphocytes [40]. Given the reduced expression of TLR genes in C3-deficient cells, one may hypothesize that cytosolic C3 translocates to the nucleus and contributes to the transcriptional regulation of these genes. For all proposed mechanisms, further investigation is essential, particularly to identify and characterize the intracellular interactors of C3. Understanding which proteins or molecular complexes interact with C3 within the cytosol or nucleus may provide critical insights into its non-canonical functions, including its role in modulating TLR expression and broader immune gene regulation. Elucidating these interactions represents a key next step in this project and may uncover novel regulatory pathways linking the complement system to intracellular immune signaling.

Taken together, our findings establish intracellular C3 as a critical regulator of innate immune signaling, revealing its unexpected capacity to fine-tune receptor expression and coordinate NF-κB-dependent cytokine responses in non-immune cells.

## Supplementary Information

Below is the link to the electronic supplementary material.

Supplementary Material 1 (XLSX 16.0 KB)

Supplementary Material 2 (XLSX 15.8 KB)

Supplementary Material 3 (XLSX 11.2 KB)

Supplementary Material 4 (XLSX 22.0 KB)

Supplementary Material 5 (PDF 1.23 MB)

## Data Availability

RNA-seq data were uploaded into Gene Expression Omnibus platform with accession number GSE301708.
