# Supplementary material for: Intracellular C3 regulates the immune response to infection via NF-κB signaling
Source: Cell Mol Life Sci. 2025 Dec 24;83(1):1. doi: 10.1007/s00018-025-05975-4 (PMC12738433; doi:10.1007/s00018-025-05975-4)
Supplement: Supplementary file 5 — Supplementary Material 5 (PDF 1.23 MB) [file 18_2025_5975_MOESM5_ESM.pdf]

Supplementary Information for 'Intracellular C3 regulates the immune response to infection via NF-kB signaling''

Katarzyna Kuska[1#], Serena Bettoni[1#], Frida Mohlin[1], Maja Chrobak[1], Vaishnavi Dandavate[1], Saleh Moradi[1], Ben King[1], Kristian Riesbeck[2] and Anna M Blom[1*].
[1] Medical Protein Chemistry and [2]Clinical Microbiology, Department of Translational Medicine, Faculty of Medicine, Lund University, Malmö, Sweden
#equal contribution
*corresponding author, anna.blom@med.lu.se

Materials and methods

RNA sequencing

Library preparation

Strand specific library
Poly-T oligo-attached magnetic beads were utilized to purify messenger RNA from total RNA. Using random hexamer primers, the first strand cDNA was synthesized post fragmentation, proceeded by second strand cDNA synthesis using dUTP, instead of dTTP. cDNA was processed by end repair, A-tailing, adapter ligation, size selection, amplification, and purification to prepare the directional library The library was analyzed by real-time PCR for quantification and Qubit and bioanalyzer for size distribution detection.

Clustering and sequencing
Following the quality assessment, different libraries were pooled according to effective concentration and targeted data amount, then subjected to Illumina sequencing.

Bioinformatics Analysis Pipeline
Data quality control
Fastp software was used for the first steps of processing raw data to clean the reads from adapters and low-quality reads. Quality metrics such as Q20, Q30, and GC content were calculated during this process. These processed reads were used for downstream analysis.

Reads mapping to the reference genome
Hisat2 v2.0.5 was used to build index of the reference genome, and paired-end clean 1 reads were aligned to the reference genome using Hisat2 v2.0.5. Reference genome and gene model annotation files were downloaded from genome website directly.

Quantification of gene expression level
FeatureCounts v1.5.0-p3 was used to count the reads numbers mapped to each gene and then FPKM of each gene was calculated based on the length of the gene and reads count mapped to this gene.

Differential expression analysis
For DESeq2 with biological replicates: Differential expression analysis was performed using the DESeq2 R package (1.44.0). The resulting P-value is adjusted using the Benjamini and Hochberg's methods to control the error discovery rate. The corrected P-value ≤ 0.05 & |log2(foldchange)| ≥ 1 was set as the threshold of significant differential expression.

KEGG enrichment analysis
KEGG enrichment was performed on significant differentially expressed genes using the clusterProfiler R package (4.12.6). Pathways containing more than 2 genes were included in the analysis.

Disease enrichment analysis
Disease enrichment was performed on significant differentially expressed genes using the DOSE R package (3.30.5).

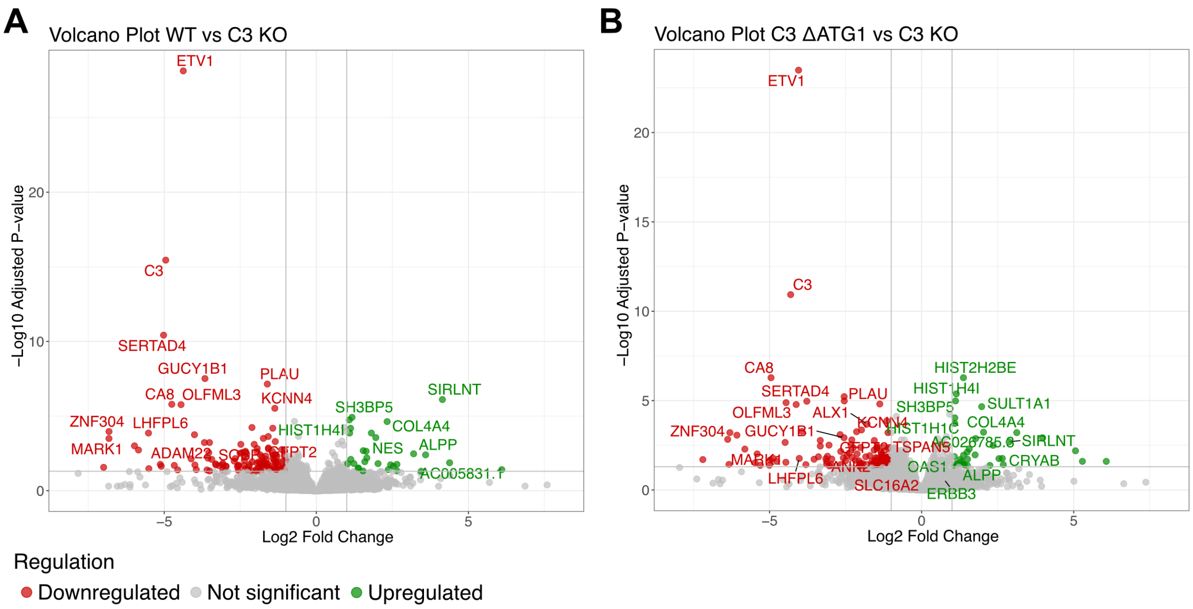

Supplementary Fig. 1 C3 deficiency alters gene expression in human lung epithelial A549 cell line, which is rescued by cytosolic C3.

Volcano plot showing many changes in gene expression (presented as $\log_2$ fold change) plotted against significance ($-\log_{10}$(padj value)) in A549 WT compared to C3 KO cells (A) and in A549 C3 KO compared to C3 ΔATG1 cells (B). Red - significantly down-regulated genes, green – significantly up-regulated (fold change >1, padj <0.05)

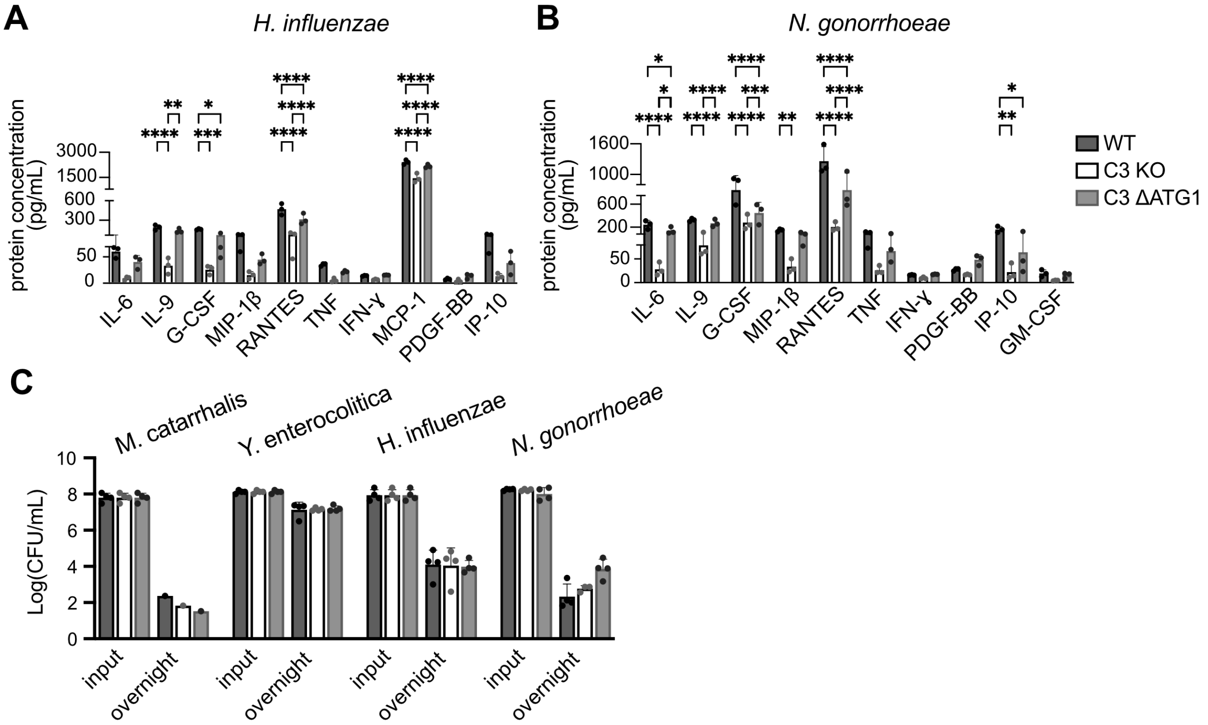

Supplementary Fig. 2 Cytokine secretion from A549 cells is modulated by the presence of C3. Bio-Plex Pro human cytokine 27-plex assay from supernatants from WT, C3 KO and C3 ΔATG1 cells infected with *H. influenzae* (A) or *N. gonorrhoeae* (B); Two-way ANOVA with Tukey´s multiple comparison test, matched measures within the experiment. Survival of intracellular bacteria after 20 hours of incubation in gentamicin-conditioned medium showed as Log(CFU/mL), C3 expression does not alter bacterial survival. Two-way ANOVA with Tukey´s multiple comparison test, matched measures within the experiment, $^{*}p < 0.05$, $^{****}p < 0.0001$

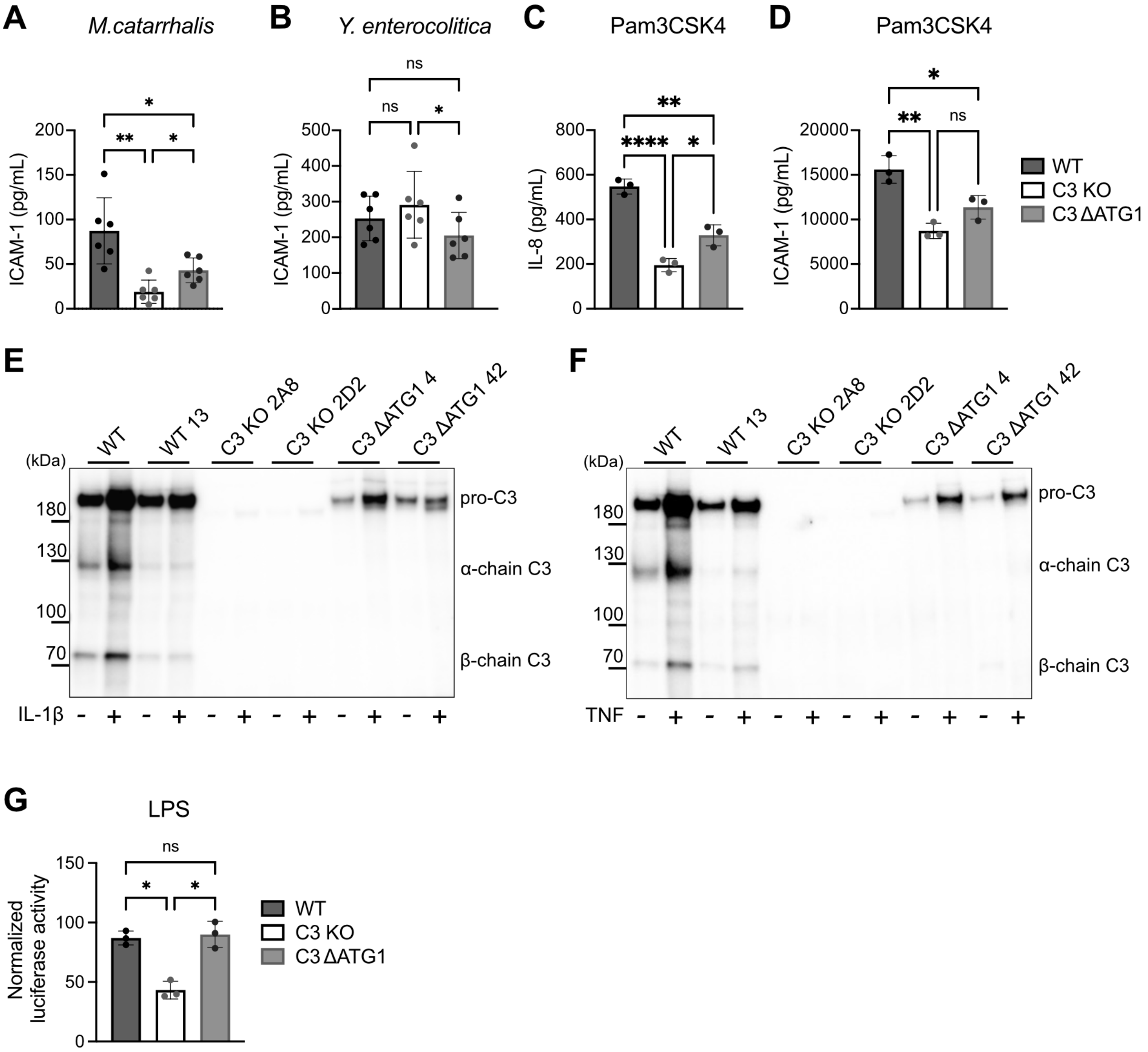

Supplementary Fig. 3 Cytokine expression from A549 cells is modulated by the presence of C3. Levels of sICAM-1 release measured by ELISA in supernatants of A549 WT, C3 KO and C3 ΔATG1 cells after infection with *M. catarrhalis* (A) are decreased in C3-deficient cells but remain unaltered after infection with *Y. enterocolitica* (B). Levels of IL-8 (C) and sICAM-1 (D) in cell lysates of A549 after Pam3CSK4 treatment measured by ELISA show downregulation in C3 KO cells. Western blots showing C3 levels in lysates of A549 WT, C3 KO and C3 ΔATG1 cells after treatment with IL-1β (E) and TNF (F) confirm upregulation of C3 following the treatment. NF-κB activation assessed using luciferase reporter assay is decreased in C3 KO compared to WT and C3 ΔATG1 cells after treatment with LPS (G). Each dot represents an average of two clones in one biological repeat. One-way ANOVA with Tukey´s multiple comparison test, matched measures within experiment. *$p < 0.05$, **$p < 0.01$, ***$p < 0.001$, ****$p < 0.0001$

Supplementary Table 1. Top 20 upstream regulators inhibited in C3 KO vs WT

Upstream regulator	Molecule type	Activation z-score	p-value of overlap
YAP1	transcription regulator	-4.586	3.79E-16
TNF	cytokine	-4.38	1.19E-39
olaparib	chemical drug	-4.248	6.02E-12
EGF	growth factor	-4.129	3.75E-37
KLF6	transcription regulator	-3.999	3.5E-10
EDN1	cytokine	-3.94	1.92E-10
STX18	transporter	-3.9	0.0000291
F2	peptidase	-3.887	9.19E-18
tetradecanoylphorbol acetate	chemical drug	-3.876	1.89E-38
NFKB (complex)	complex	-3.829	5.97E-18
WNT3A	cytokine	-3.704	3.83E-16
IGE (complex)	complex	-3.692	2.64E-14
HMG20A	transcription regulator	-3.657	2.12E-10
IL1B	cytokine	-3.59	1.33E-33
CYTOKINE (family)	group	-3.504	0.00000027
poly rI:rC-RNA	biologic drug	-3.494	5.61E-12
TLR3	transmembrane receptor	-3.416	0.0000947
TP53	transcription regulator	-3.395	1.57E-17
FTO	enzyme	-3.392	1.53E-16
SMAD3	transcription regulator	-3.375	1.66E-11

Supplementary Table 2 Top 20 upstream regulators inhibited in C3 KO vs C3 ΔATG1

Upstream regulator	Molecule type	Activation z-score	p-value of overlap
TNF	cytokine	-6.03	3.04E-30
EGF	growth factor	-4.885	7.46E-31
F2	peptidase	-4.769	5.79E-20
IL4	cytokine	-4.663	3.19E-17
AGT	growth factor	-4.605	1.68E-19
STX18	transporter	-4.583	0.000012
tetradecanoylphorbol acetate	chemical drug	-4.565	9.35E-25
NFKB (complex)	complex	-4.562	2.84E-15
IGE (complex)	complex	-4.449	1.91E-09
RELA	transcription regulator	-4.365	5.79E-13
IL1B	cytokine	-4.276	2.67E-21
KLF6	transcription regulator	-4.09	1.06E-11
poly rI:rC-RNA	biologic drug	-4.019	8.61E-13
SMARCA4	transcription regulator	-3.972	1.65E-18
NUPR1	transcription regulator	-3.952	1.36E-10
HMG20A	transcription regulator	-3.92	9.54E-11
olaparib	chemical drug	-3.91	1.16E-10
KCNJ2	ion channel	-3.9	0.000426
FGF2	growth factor	-3.85	8.18E-15
EDN1	cytokine	-3.813	2.6E-14
